# Co-Enriching Microflora Associated with Culture Based Methods to Detect *Salmonella* from Tomato Phyllosphere

**DOI:** 10.1371/journal.pone.0073079

**Published:** 2013-09-09

**Authors:** Andrea R. Ottesen, Antonio Gonzalez, Rebecca Bell, Caroline Arce, Steven Rideout, Marc Allard, Peter Evans, Errol Strain, Steven Musser, Rob Knight, Eric Brown, James B. Pettengill

**Affiliations:** 1 Molecular Methods and Subtyping Branch, Division of Microbiology, Office of Regulatory Science, Center for Food Safety and Applied Nutrition, United States Food and Drug Administration, College Park, Maryland, United States of America; 2 Biofrontiers Institute, University of Colorado, Boulder, Colorado, United States of America; 3 Virginia Tech, Virginia Agricultural Experiment Station, Painter, Virginia, United States of America; 4 Biofrontiers Institute, University of Colorado, Boulder, Colorado, United States of America; 5 Department of Chemistry and Biochemistry, University of Colorado, Boulder, Colorado, United States of America; 6 Howard Hughes Medical Institute, Boulder, Colorado, United States of America; Cornell University, United States of America

## Abstract

The ability to detect a specific organism from a complex environment is vitally important to many fields of public health, including food safety. For example, tomatoes have been implicated numerous times as vehicles of foodborne outbreaks due to strains of *Salmonella* but few studies have ever recovered *Salmonella* from a tomato phyllosphere environment. Precision of culturing techniques that target agents associated with outbreaks depend on numerous factors. One important factor to better understand is which species co-enrich during enrichment procedures and how microbial dynamics may impede or enhance detection of target pathogens. We used a shotgun sequence approach to describe taxa associated with samples pre-enrichment and throughout the enrichment steps of the Bacteriological Analytical Manual's (BAM) protocol for detection of *Salmonella* from environmental tomato samples. Recent work has shown that during efforts to enrich *Salmonella* (Proteobacteria) from tomato field samples, Firmicute genera are also co-enriched and at least one co-enriching Firmicute genus *(Paenibacillus* sp.) can inhibit and even kills strains of *Salmonella*. Here we provide a baseline description of microflora that co-culture during detection efforts and the utility of a bioinformatic approach to detect specific taxa from metagenomic sequence data. We observed that uncultured samples clustered together with distinct taxonomic profiles relative to the three cultured treatments (Universal Pre-enrichment broth (UPB), Tetrathionate (TT), and Rappaport-Vassiliadis (RV)). There was little consistency among samples exposed to the same culturing medias, suggesting significant microbial differences in starting matrices or stochasticity associated with enrichment processes. Interestingly, *Paenibacillus* sp. (*Salmonella* inhibitor) was significantly enriched from uncultured to cultured (UPB) samples. Also of interest was the sequence based identification of a number of sequences as *Salmonella* despite indication by all media, that samples were culture negative for *Salmonella*. Our results substantiate the nascent utility of metagenomic methods to improve both biological and bioinformatic pathogen detection methods.

## Introduction

Enrichment protocols to detect pathogens of interest vary from pathogen to pathogen and depending on matrix of origin. The Bacterial Analytical Manual (BAM) produced by the United States Food and Drug Administration (FDA) describes methods for isolating *Salmonella* ssp. from more than 25 different matrices (e.g., leafy greens, tomatoes, eggs, etc.). The level of specialization required to isolate the same species from different matrices illustrates the complexities associated with culturing methods for detecting pathogens. The substantial number of enrichment procedures also suggests that no single method is superior to others in all cases.

Despite these levels of specialization, it is often even difficult to detect pathogens from samples that have been intentionally spiked. For example, average sensitivity across three different culturing techniques for detection of *Salmonella enterica* from tomato samples with known contamination was approximately 77% [1]. Work by Gorski [2] demonstrated that for currently unknown reasons, some serotypes of *Salmonella* appear to be more fit in certain media, which could clearly bias certain investigations by favoring recovery of serovars with less relevance to outbreaks. These studies and others [3] suggest that serotype recovery is likely biased in many situations and that we may fail to recover pathogens of importance more often than we realize. Explanations for some biases are likely correlated with the currently un-described microbial dynamics associated with the complex assemblages of microflora that co-culture during efforts to culture target pathogens.

Published [3], and unpublished work by FDA scientists has repeatedly demonstrated that during efforts to culture *Salmonella* and other target Enterobacteriaceae pathogens within Proteobacteria, Firmicute genera are co-enriched. This phenomenon has been observed numerous times from cultures of phyllosphere and soil samples. Of particular interest is the fact that one co-enriching Firmicute genus, *Paenibacillus* has been shown to inhibit and reportedly kill *Salmonella.* A patent has even been filed for a newly isolated non-pathogenic bacterial strain of *Paenibacillus*, known as TS-15 which has shown the ability to kill or inhibit a wide variety of harmful bacteria including many of the most common food-borne pathogens such as *Salmonella*, *Escherichia*, *Listeria*, *Shigella*, *Enterobacter* and *Staphylococcus* (www.ott.nih.gov/Technologies/abstractDetails.aspx?RefNo=2396). This type of information is obviously significant for the streamlining of efforts to culture specific pathogenic genera and strains from diverse food, environmental and biological matrices.

For decades, targeted amplification of taxon-specific DNA regions has been useful for pathogen detection, but recently whole genome sequencing (WGS) has become an affordable method for analyzing pathogens with small genomes. Compared to multi-locus PCR targets or restriction digest methods such as PFGE, WGS allows us to investigate outbreaks with an unprecedented degree of resolution [4], [5]. However, even WGS methods currently require pre-enrichment procedures to obtain isolates for sequencing, raising again, the importance of better understanding culturing dynamics.

A goal for pathogen detection that lies ahead, is the use of metagenomic approaches to identify pathogens directly from sequence data, independent of any culturing steps at all. Culture free methods do not suffer biases introduced by enrichment procedures [3], [6]–[9]. While culture independent metagenomic approaches have already proved useful, as in the case of the recovery of the draft genome of the outbreak strain of Shiga-Toxigenic *Escherichia coli* O104:H4 from fecal samples (Loman et al. 2013), there are still limitations associated with this approach. These include; the very low number of target pathogen cells, the vast quantity of sequence data needed to sequence a specific strain from amidst a complex microbial community, incomplete reference databases for most environments and intensive computational requirements for the processing of very large datasets (180 GB were generated by Loman et al.). A useful intermediate step may be the use of shotgun sequencing in association with culturing procedures and application of bioinformatic detection methods to identify pathogens directly from enrichments. The work presented here was designed to explore the utility of a metagenomic approach to describe the complex mileu of microflora that co-enriches (assuming the target is enriching) during the BAM protocol for detection of *Salmonella* from tomato phyllosphere samples. These data will assist with future efforts to improve culturing methods and bioinformatic detection of pathogens directly from metagenomic and shotgun sequenced enrichments. We examined DNA from four uncultured phyllosphere replicates and followed these samples through three enrichment steps used in the BAM for detection of *Salmonella.* The three culturing conditions were: 1) Universal Pre-enrichment broth (UPB) which provides buffering against rapid changes in pH to aid growth of sublethally-injured *Salmonella* [10]; 2) Rappaport-Vassiliadis (RV) more selective, with low pH, Malachite Green, and high MgCl_2_ to increase osmotic pressure, [11], [12]; and 3) Tetrathionate (TT) broth, also selective for *Salmonella* spp. by reported suppression of commensal intestinal organisms, with a combination of Sodium Thiosulfate (Na_2_S_2_O_3_) and tetrathionate [13].

Another goal of this study was to examine the efficiency of different bioinformatic classification pipelines and approaches such as assembly vs. no-assembly, to describe taxonomic profiles associated with BAM enrichments for detection of *Salmonella* from tomato phyllosphere. A vitally important future step for the validation of this methodology will be the addition of known concentrations of *Salmonella* (or other target pathogens) to samples. This will allow us to determine the amount of sequence data necessary for bioinformatic recovery of an introduced strain as well as improve our understanding of the biological dynamics that may be associated with inhibition by co-culturing organisms.

## Materials and Methods

### Sample collection and enrichment

Tomato phyllosphere samples were collected from the Eastern Shore Agricultural Research and Extension Center of Virginia Tech, in Painter, VA on July 15, 2011. No specific permissions were required for collection from these research fields other than the consent of the Virginia Tech agricultural research scientists and extension agents who direct the activities of this Virginia Agricultural Experiment Station. The field studies did not involve endangered or protected species. Four independent samples were collected with each sample comprised of ten leaves and four tomatoes picked randomly from different plants in a single row approximately 23 meters in length. Each of the four bags of pooled leaves and tomatoes came from a different row – in an attempt to get a broad representation of the field. Samples were stored at 4°C for 48 hours. Subsets of the four samples were used to create the uncultured (UNC) treatment. Sterile water (300 ml) was added to each phyllosphere subset (5 leaves and 2 tomatoes). Bags were sonicated to disrupt biofilms associated with leaf and fruit surfaces and the resulting “wash” water was centrifuged and DNA was extracted from the pellet. The remaining samples were enriched in UPB overnight and aliquots of the UPB enrichment were added to TT and RV medias. Approximately one ml of each enrichment was collected after the 24 hour incubation period and pelleted for DNA extraction (Table 1). A real time PCR assay developed for detecting *Salmonella* [14] was also performed on UPB enrichments. No positive real time results were observed for any of the samples.

**Table 1:** MG-RAST ID, BioSampleID, total number of bases (Yield), and average length of sequences per sample, of the 15 metagenomes for both the FLASH and Meta-Velvetg assembly methods.

Treatment	Sample ID	MG-RAST ID			Yield (Mbp)		Length (average)	
		FLASH	Meta-Velvetg	BioSampleID	FLASH	Meta-Velvetg	FLASH	Meta-Velvetg
Uncultured	UNC1	4502820	4502821	SAMN01760734	21.43	1.93	205	179
	UNC2	4502822	4502823	SAMN01760735	10.21	0.94	202	175
	UNC3	4502824	4502825	SAMN01760736	63.16	7.07	209	168
Rappaport-Vassiliadis	RV1	4502804	4502805	SAMN01760726	65.8	2.52	187	185
	RV2	4502806	4502807	SAMN01760727	176.71	18.23	212	235
	RV3	4502808	4502809	SAMN01760728	634.61	5.55	207	1574
	RV4	4502810	4502811	SAMN01760729	163.09	3.74	185	186
Tetrathionate	TT1	4502812	4502813	SAMN01760730	56.59	4.99	213	217
	TT2	4502814	4502815	SAMN01760731	84.43	5.5	229	595
	TT3	4502816	4502817	SAMN01760732	96.91	4.5	214	1508
	TT4	4502818	4502819	SAMN01760733	121.12	18.32	221	234
Universal Pre-Enrichment Broth	UPB1	4502826	4502827	SAMN01760737	128.73	17.71	219	209
	UPB2	4502828	4502829	SAMN01760738	271.52	14.56	214	507
	UPB3	4502830	4502831	SAMN01760739	312.66	14.32	221	328
	UPB4	4502832	4502833	SAMN01760740	267.26	11.86	218	405
	Average				164.95	8.78	210	447

### DNA extraction, library preparation and DNA sequencing

DNA was extracted from cultured and culture independent samples using the Promega Wizard® Genomic DNA purification Kit (Promega Corporation, Madison, WI) following the extraction protocol for Gram-negative bacterial species. We used 50 ng of DNA from each replicate as input for the Nextera DNA Sample Preparation Kit (Illumina, San Diego, CA) with the Associated Nextera Index Kit according to the manufacturers specifications. Libraries were diluted to 2 nM and denatured with.1N NaOH according to Illumina's specification for sequencing on the MiSeq V1 platform that produces 2×151 reads.

### Sequence assembly

We performed two different steps that increased the length of reads and therefore should have increased our ability to accurately assign taxonomy and function. The first approach was to use the Fast Length Adjustment of Short reads (FLASH) [15] program that combines paired-end reads that overlap into a single contig. We used the default settings that included 10 bp minimum overlap between reads.

In the second approach, we performed de novo assemblies of the metagenomes using Meta-Velvet [16], which uses the program velveth to construct *k*-mer hash tables and the program velvetg to construct an initial de Bruijn graph [17]. Next, the Meta-Velvetg program decomposes the initial de Bruijn graph into sub-graphs from which contigs were built representing the different genomes in the sample. Although this process loses the information about the abundance of particular taxa, these longer reads may increase the taxonomic resolution that can be assigned to individual reads. (Abundance data can be regained later by blasting the original reads back to the assembled contigs).

### Taxonomic classification

To determine how the different culturing techniques altered the taxonomic profiles of the samples, we used the reference-based approach implemented within MG-RAST [18] that utilizes the M5 non-redundant database (M5NR), a compilation of many databases (e.g., BLAST nr, KEGG, and Uniprot). It is important to note that by assigning taxonomy based on translated nucleotide – protein homology we lose information contained in the 10–20% of microbial genomes that are not protein coding [19] and are unable to account for lineage specific differences in codon bias [20]. We classified reads based on the lowest common ancestor approach, which assigns each read the taxonomy of the lowest taxonomic rank among the best hits. For all analyses in MG-RAST we used a maximum e-value cutoff of 1.0^−5^, minimum percent identity of 95%, and minimum alignment length of 33 amino acids (99 bp; MG-RAST classifications are based on amino acid similarity). Overall taxonomic differences were estimated through construction of a Principal Coordinates Analysis (PCoA) based on normalized Bray-Curtis distances. To account for differences in the number of reads among the samples, we present differences in the normalized abundances of different taxonomic groups. We performed paired *t*-tests using R [21] to determine whether there were significant differences between the different enrichments and the control (uncultured).

### Pathogen detection pipeline for *Salmonella*

We used a novel pipeline, found in https://github.com/qiime/platypus, that was developed to detect a specific organism, in this case, *Salmonella*. The classification approach used sequences from the Integrated Microbial Genomes (IMG) database and scripts from the Quantitative Insights Into Microbial Ecology (QIIME) package to construct a pair of databases. The first, labeled InterestDB, contained only known *Salmonella*-specific sequences, and the second, labeled OtherDB, consisted solely of non-*Salmonella*. Sequences were quality-filtered (split_libraries.py) and then analyzed using the program parallel_blast.py with an extremely liberal setting (i.e., E-value = 0.1) against InterestDB and against OtherDB to maximize the number of hits to each database. We then ran the platypus_compare.py, which, as the name suggests, compares the BLAST results against each database and returns the better hit from the two databases. The parameter settings for this step are much more stringent (i.e., E-value = 1^−30^) and we evaluated a number of different percent identity and percent overlap thresholds. We ran the analyses using 100% identity across at least 100 bp. The best hit for a given sequence was determined by the BLAST result for those parameters that had the best bit score between the two databases. To determine the gene regions to which these putative *Salmonella* reads belonged, we BLASTed them, using the same criteria, against an FDA in-house collection of 156 annotated *Salmonella* genomes.

We were also interested in estimating the percentage of species within a sample that we did not detect and how much more sequence data (i.e., bps) we would have needed to obtain approximately 1X coverage across all taxa within a sample. To accomplish the former, based on the FLASHed results we estimated the additional number of OTUs that would have been observed given additional sampling based on the Solow estimate using the calculation in MOTHUR [22]. We calculated the Solow estimate based on if we had double the amount of sequences per sample (the estimate is only valid when the additional number of reads is equal to or less than those actually obtained). To estimate the number of bases necessary to achieve 1X coverage across all genomes, we assumed that the average genome size was 5 Mbp that we then multiplied by the total number of species observed. We then compared this to the number of bp we actually acquired. We acknowledge that this a simplistic approach, but feel that it represents a significant underestimate of the actual number of bp we would have needed. As a result, such information can serve as a conservative heuristic regarding the additional sequencing effort necessary to assemble the genomes of taxa present in an environmental sample. This estimate was also based only on the FLASHed reads.

### Functional classification

MG-RAST was also used to evaluate whether functional differences (i.e., the presence or absence of particular genes) could be observed among the different culturing techniques. We used protein databases such as COG, NOG, SEED, and KEGG that hierarchically group proteins. We focused on differences based on classification of reads to the highest level in the functional hierarchy (i.e., Level 1). The same search parameters were used as those in the taxonomic classification. A PCoA plot was then constructed based on normalized Bray-Curtis distances.

## Results

### Sequencing yield

After assembling overlapping MiSeq reads with FLASH, we obtained 15 million sequences totaling 2.6 Gbp (Table 1). *De novo* assembly using Meta-Velvetg resulted in many fewer sequences; 3.1×10^4^ reads, compared to 9.9×10^5^ for FLASHed reads. As expected, Meta-Velvetg reads were longer (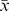 = 447 bp) than the FLASHed reads (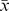 = 210 bp). However, it is important to note that the *de novo* assembly was done on reads that had not been merged, therefore the Meta-Velvetg reads are, on average, shorter than the FLASHed reads for some samples (Table 1). We attribute the short mean fragment size relative to the maximum (290 bp) for the FLASHed reads to an insert size of greater than 302 bp (2×151). All metagenomes are publicly available from MG-RAST and SRA at NCBI, see Table 1 for all accession numbers and BioSampleIDs.

### Taxonomic descriptions of metagenomic and shotgun sequenced enrichments

**Figure 1:**
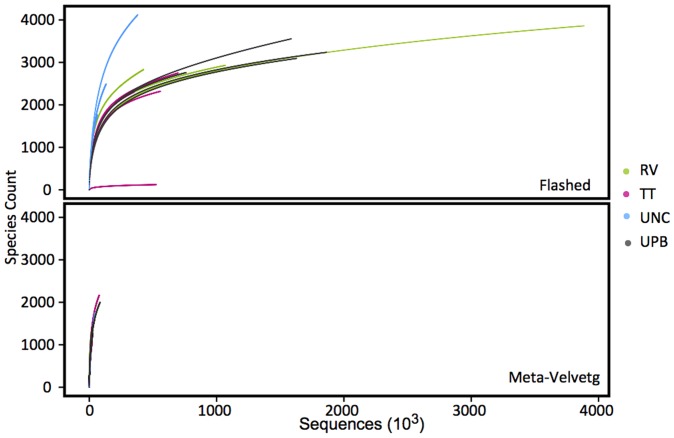
Rarefaction plots illustrating the increase in taxa identified as a function of different sequencing depths for a) flashed and b) Meta-Velvetg reads.

Rarefaction plots of species diversity as a function of number of FLASHed reads generally showed that the sequencing depth was insufficient to capture the majority of the diversity within our samples (Fig. 1a). This pattern was even more pronounced when using Meta-Velvetg assembled reads, which did not come close to asymptote (Figure 1b). This is not surprising, due to the reduced abundance of reads following assembly. The rarefaction plots also indicated that species diversity was greater within the FLASHed samples compared to the Meta-Velvetg assembled reads. Based on the Solow estimate of the additional number of species we would have detected if we had obtained double the number of reads, we would still have only sampled 83% of the species present at extremely low coverage.

**Figure 2:**
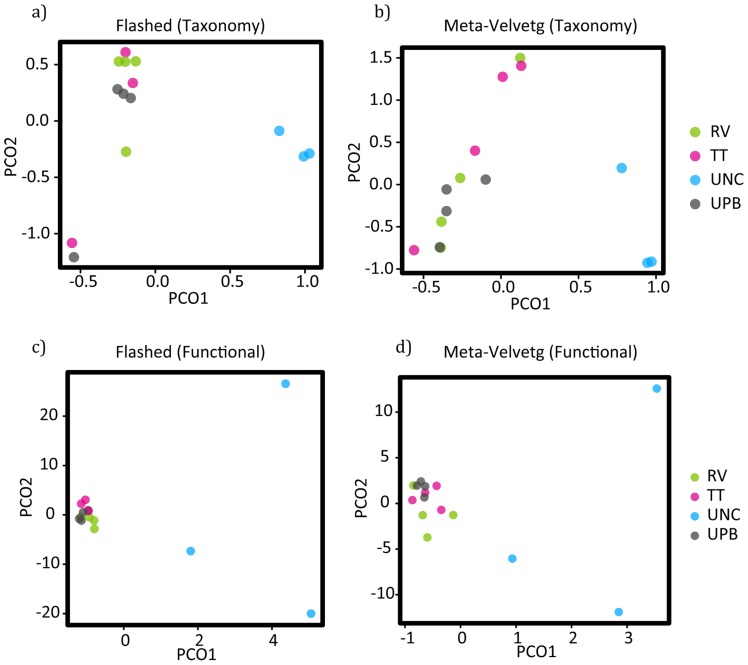
Principal Coordinates Analysis (PCoA) depicting the taxonomic (a and b) and functional (c and d) differences among the replicates and treatments.

Based on PCoA plots of both Flashed and Meta-Velvetg reads – it is not surprising that uncultured (UNC) samples were the most diverse in terms of their overall taxonomic profile. The UNC samples clustered together independently of the enriched samples along axis 1 (Fig. 2). Although there was some differentiation among samples with respect to Axis 2, there did not seem to be any clear separation by culturing technique (UPB, TT, RV). Data from samples from the same enrichment medias did not cluster together – perhaps because they were not laboratory replicates but rather independent field replicates with inherent beta-diversity. To make sure the observed patterns were not relics of insufficient or unbalanced sampling, we reran the PCoA analyses on a rarefied subset of the data and observed the same pattern (Figure S1).

**Figure 3:**
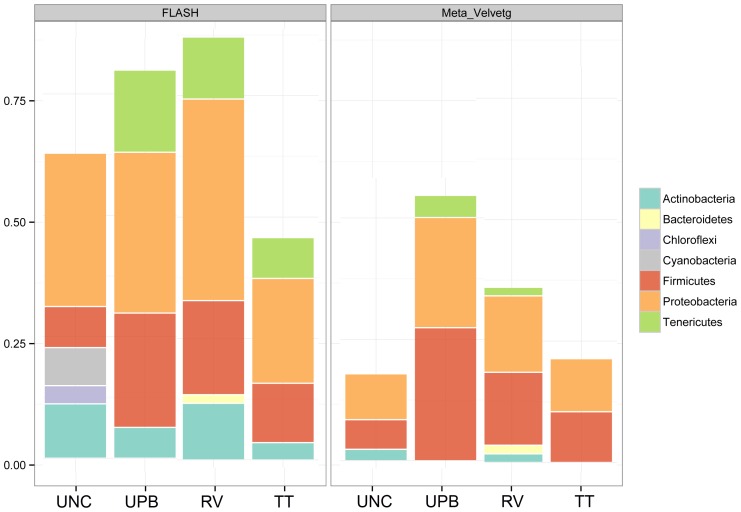
Taxonomic description of Phyla in uncultured and cultured samples using Flash and Meta Velvetg for assembly and the lowest common ancestor taxonomic rank of best hits (maximum e-value cutoff of 1.0^−5^, minimum percent identity of 95%, and minimum alignment length 99 bp).

**Figure 4:**
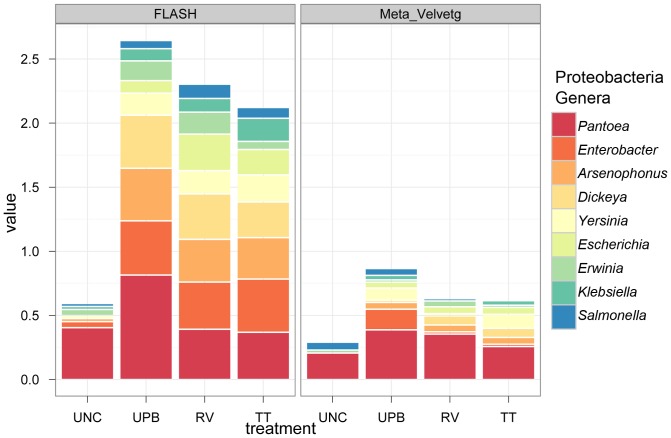
Taxonomic classification of the nine most prevalent Proteobacteria genera in uncultured and cultured samples based on FLASHed and Meta Velvetg assembly and the lowest common ancestor taxonomic rank among the best hits (maximum e-value cutoff of 1.0^−5^, minimum percent identity of 95%, and minimum alignment length 99 bp).

In general, a greater number of sequences could be assigned taxonomy from the FLASHed samples compared to the assembled Meta-Velvetg raw reads (Table 1). This result is in part driven by higher abundances of specific taxa within the FLASHed samples, which is to be expected, as multiple copies of the same region will be collapsed into a single contig using Mega-Velvetg. There were also instances where taxa detected with the FLASHed samples were not present in the Mega-Velvetg samples (Table 1 and Figs. 3 and 4). Observed Phyla across the samples included Actinobacteria, Tenericutes, Chloroflexi, Cyanobacteria, Bacteriodetes, Cyanobacteria, Proteobacteria and Firmicutes (Figure 3). All of which have been reported numerous times associated with the phyllosphere [23]–[26].

**Figure 5:**
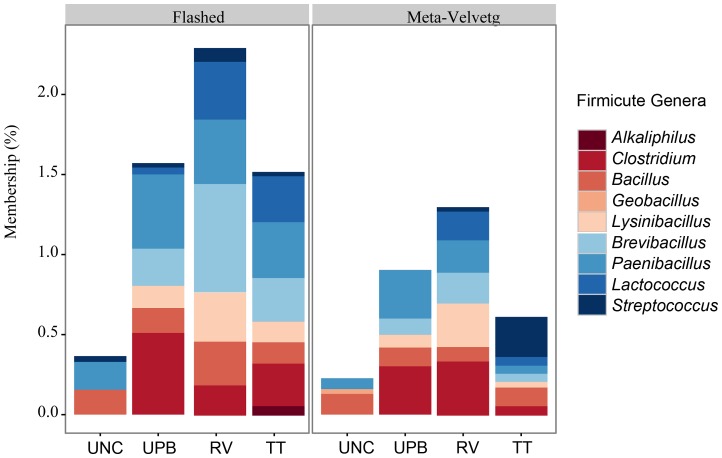
Taxonomic classification of Firmicute genera associated with metagenomic and shotgun sequenced enrichments using FLASHed and Meta Velvetg assembly and the lowest common ancestor taxonomic rank among the best hits (maximum e-value cutoff of 1.0^−5^, minimum percent identity of 95%, and minimum alignment length 99 bp).

**Figure 6:**
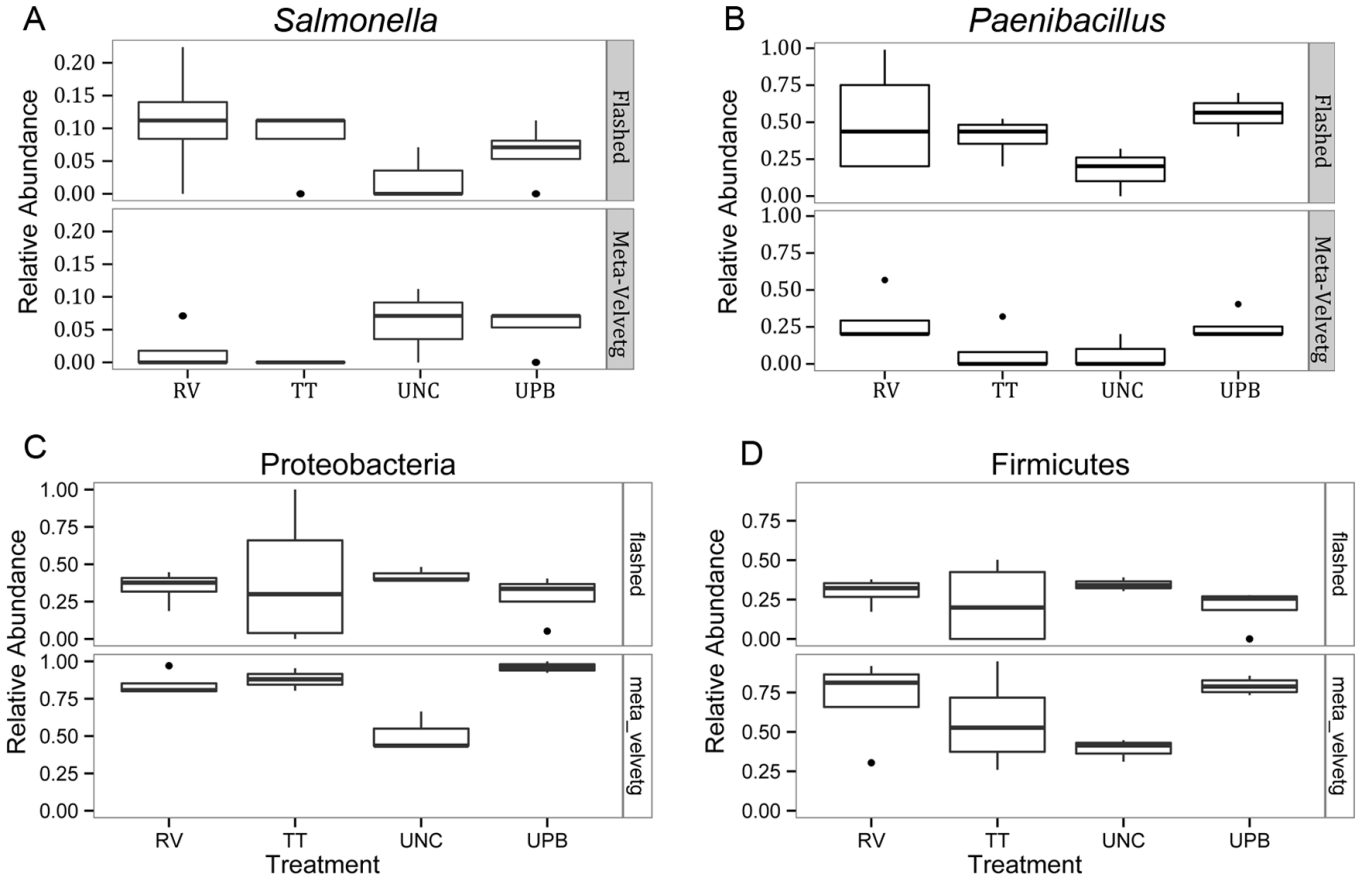
Boxplots of the relative abundance of a) *Salmonella*, b) *Paenibacillus* c) Proteobacteria, and d) Firmicutes among the different treatments using the FLASHed and Meta Velvetg assemblies. Boxes show the interquartile range, bars illustrate the median, and the whiskers extend out to 1.5 times the interquartile range.

Within the Firmicutes, the most prominent genera were *Clostridium, Bacillus, Brevibacillus, Paenibacillus* and *Lactococcus* (Fig. 5). As expected, an abundance of Firmicutes and Proteobacteria was observed in all samples, including uncultured (Figs. 4 and 6). Significant differences in the abundance of *Paenibacillus* sp. (*Salmonella* inhibitor) were observed between the uncultured and UPB treatments. However, no significant differences in the abundances of *Paenibacillus* sp. were observed among the different medias (*P*<0.05). The UPB and RV enrichments had the highest abundance of Firmicutes and Proteobacteria for both FLASHed and Meta-Velvetg reads (Fig. 6).

Dominant observed Proteobacteria genera were *Pantoea*, *Enterobacter, Dickeya*, and *Arsenophonus* (Table 2, Figs. 4). *Pantoea* was the dominant taxonomic group across all treatments, although this was likely only true for uncultured samples due to the inability of the majority of the reads to be mapped to any genera (Fig. 4). Statistically significant differences in the abundance of sequences from uncultured to cultured treatments were observed for *Enterobacter*, *Dickeya* and *Arsenophonus* using FLASHed reads (Fig. 4).

**Table 2:** Number of reads classified based on a lowest common ancestor approach to bacterial genera across the samples and different assembly methods.

	Flashed				Meta-Velvetg			
Genus	UNC	TT	RV	UPB	UNC	TT	RV	UPB
*Bacillus*	41	2511	17296	26580	3	834	3499	2064
*Pantoea*	652	2680	5076	21914	86	837	459	1943
*Acinetobacter*	0	159	2958	3780	0	21	308	36
*Enterobacter*	1	881	279	295	0	65	5	16
*Clostridium*	0	22	652	520	0	1	96	24
*Lysinibacillus*	0	3	554	2	0	1	182	16
*Streptococcus*	0	429	2	4	0	155	2	0
*Lactococcus*	0	93	154	25	0	22	26	0
*Arsenophonus*	0	31	94	154	0	1	1	1
*Escherichia*	0	34	227	13	0	4	1	1
*Paenibacillus*	1	23	77	77	0	3	4	7
*Dickeya*	0	30	88	68	0	1	1	0
*Pseudomonas*	1	133	6	5	0	0	1	1
*Erwinia*	1	4	34	55	0	0	1	0
*Brevibacillus*	0	4	39	9	0	1	3	1
*Salmonella*	0	41	8	3	1	0	0	1
*Yersinia*	0	19	13	8	0	3	0	4
*Exiguobacterium*	0	2	33	9	0	1	1	1
*Acholeplasma*	0	11	9	23	0	0	0	1
*Klebsiella*	0	18	9	10	0	1	0	1
*Acetobacter*	0	18	13	7	0	0	0	0
Candidatus_*Regiella*	0	2	6	23	0	0	0	1

Results are shown in rank order and for only those genera with greater than 30 reads assigned to them.

### Salmonella detection

**Figure 7:**
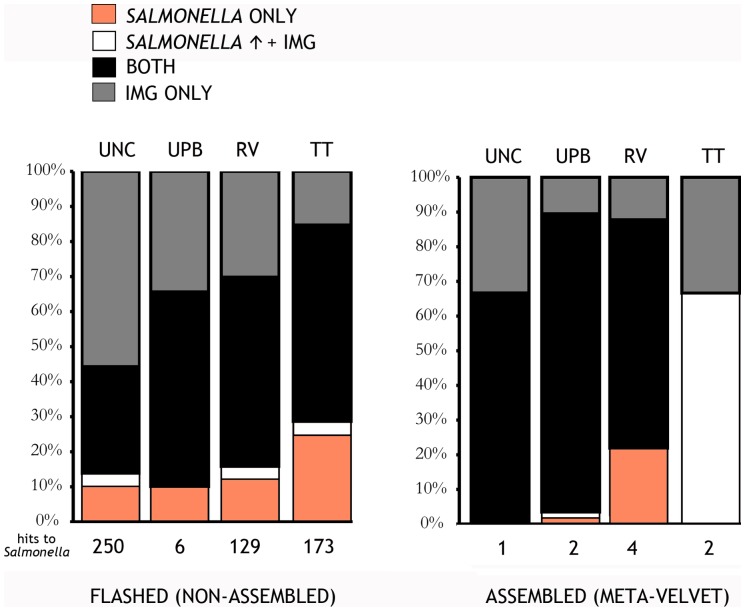
Results of the IMG pipeline assigning reads to either only *Salmonella* (*Salmonella* Only, orange), both *Salmonella* and the other database but with greater confidence to the former (Salmonella ↑ + IMG, white), both databases with equal confidence (both, black), or the other database only (IMG Only, grey) for a) flashed and b) Meta-Velvetg reads.

All RT-PCR results from BAM enrichment procedures for these samples were negative, however results from the bioinformatic pipeline showed putative hits to *Salmonella*. The hits were based on the pipeline described in Materials and Methods, (division of the IMG database into InterestDB (*Salmonella*) and OtherDB (all other taxa)) and a comparison of hit scores at ascending thresholds. Among all treatments, TT had the highest incidence followed by RV, UPB, and lastly the uncultured treatment (Fig. 7) – which in itself, supports the potential legitimacy of these hits (because hits would be more likely among cultured samples). Further exploration of the putative hits revealed that they often matched to more than one *Salmonella* serovar (e.g., *S.* Agonoa, *S.* Newport, and *S.* Montevideo). They were also predominantly associated with ribosomal genes, which is not be surprising given the higher copy number of rRNA genes, but this fact certainly lessens the diagnostic significance of these hits. Interestingly, FLASHed reads tended to have a higher number of putative *Salmonella* than Meta-Velvetg reads. Although more putative *Salmonella* sequences were observed within the enriched samples, there was not a statistically significant difference when compared to the uncultured samples. Specifically, *P* = 0.143 between the uncultured and UPB samples, *P* = 0.080 between the uncultured and TT treatments, and *P* = 0.077 between uncultured and RV samples.

### Functional differences

AS with the taxonomic differences, we found that the uncultured samples were distinct from enrichment treatments in the abundance of different functional groups (Fig. 2). We also found overlap among replicates from the same enrichment treatment along Axis 2 of the PCoA plot. Axis 1 predominantly differentiates uncultured samples from the enrichment samples.

## Discussion

Using a metagenomic approach, we examined the microflora of samples pre and post enrichment to describe non-target microbial species that co-culture during enrichment steps associated with the BAM for isolation of *Salmonella* from tomato phyllosphere samples. We provided a preliminary taxonomic survey of organisms pre-enrichment and a preliminary survey of taxonomy in response to enrichments. These data will assist with estimates of the depth of sequencing that will be needed for diagnostics associated with cultured and uncultured phyllosphere microflora. They also provide estimates of optimal bioinformatic approaches (e.g., assemble or not) required to reliably detect a pathogen from a metagenomic or shotgun sequenced sample. Although our results clearly demonstrate that the different enrichment methods investigated had significant effects on the taxonomic profiles of the samples relative to controls, they also suggested that there may be a degree of stochasticity in enrichment procedures. Laboratory replicates in addition to field replicates should be added to subsequent experiments to better address this question. However, the fact that the uncultured samples clustered together, suggests that the independent field replicates were comprised of similar microbial consortia. Rarefied subsets of the data did not produce a different PCoA pattern (SuTherefore, the possibility exists that laboratory replicates will not always produce the same taxonomic profiles in terms of presence/absence or abundance post-enrichment due to currently un-described microbial dynamics. This pattern was less pronounced for functional differences: all enrichment procedures appeared to select similar functional groups with little variation among replicates including uncultured replicates. The fact that the majority of the reads from the uncultured tomato phyllosphere were assigned to only a few genera, *Pantoea* and *Bacillus*, is likely not indicative of low diversity, but rather an artifact of the inability to assign taxonomy to many of the reads at the relatively stringent criteria selected, because the majority of species within these samples are not well-represented in existing databases [27], [28]. Of particular importance is the possibility that we detected *Salmonella* based on two conservative methods using shotgun metagenomics when PCR and culture techniques were unable to do so. The future addition of experiments that include the spiking of known concentrations of *Salmonella* will be crucial to validate these results and guide future metagenomic and biological culture based detection methods.

**Figure 8:**
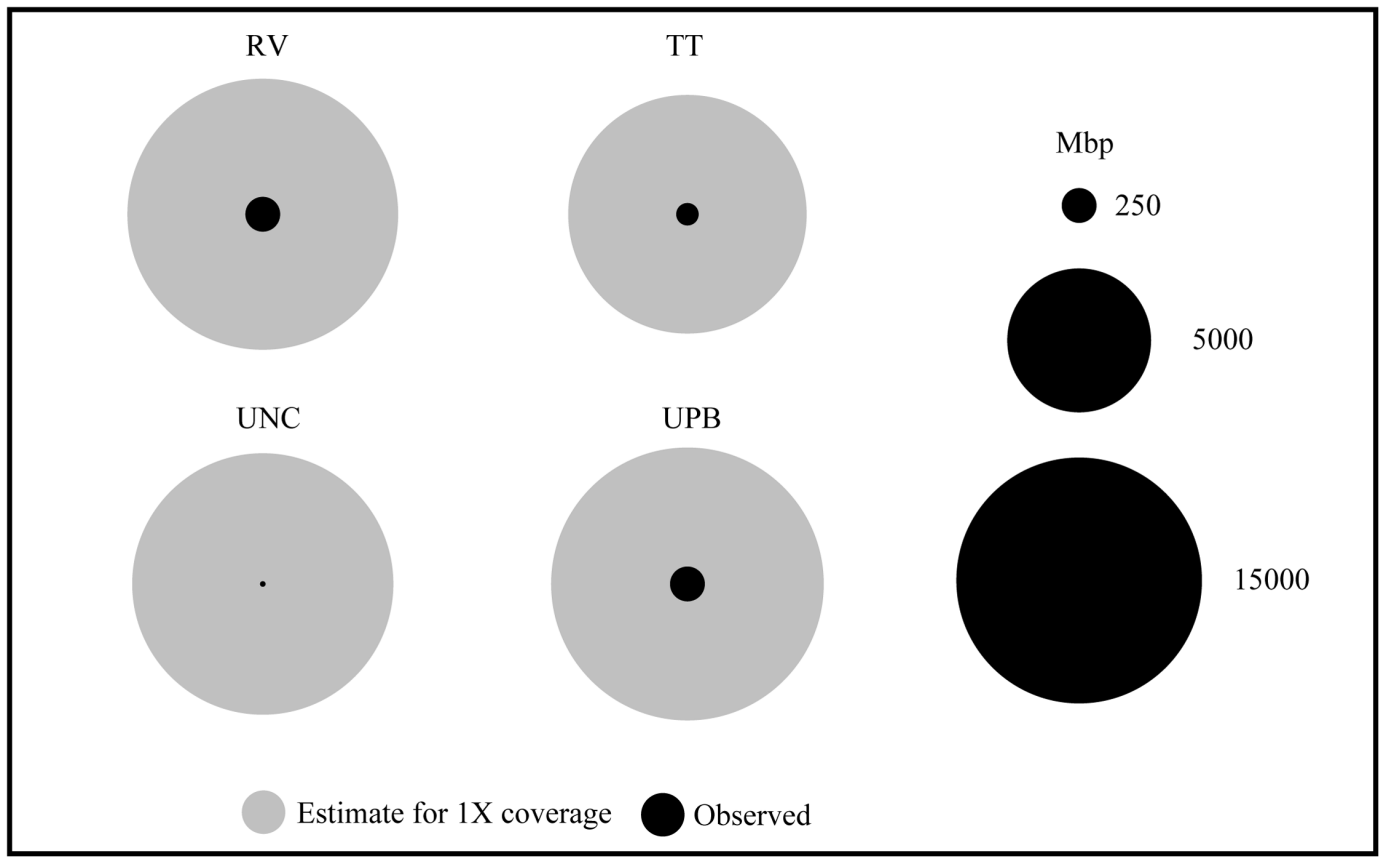
Plots of the average number of base-pairs (in millions) observed and estimates of quantity necessary to achieve approximately 1X coverage across all genomes present in cultured and uncultured samples.

The future of metagenomics as a diagnostic tool for detecting pathogens rests in large part on several criteria: fraction of genomes present that are sequenced (coverage), read length (perhaps less important), completeness of reference databases, and computational power. Our results suggest that we have extremely low coverage across many genomes in that only tens to hundreds of 151 bp reads were assigned to many taxa with genomes sizes around 5 Mb. Based on our simplistic estimate, we would have needed to acquire on average approximately 250 times as many bp to achieve 1X coverage across all genomes present in a given replicate (Fig. 8).

As for read length, we analyzed two different bioinformatic approaches to increase the length of contigs and, thus, increase our ability to assign taxonomy. These two approaches did increase the average read length beyond a single paired-end read (e.g., assemblies were greater than 151 bp; average for FLASHed and Meta-Velvetg reads were 210 and 450 bp, respectively; Table 1). However, a comparison of those results is somewhat surprising: increased read length reduced our ability to assign taxonomy. In some instances we actually lost the ability to detect a species by merging reads based on the Meta-Velvetg approach (e.g., UPB treatment and the detection of *Lactococcus* and *Dickeya*; Table 2). As a result, it does not appear that read length may be the most significant impediment to classification and detection. Our results and those of others [29] raise an interesting question of whether there is much to be gained from performing *de novo* assemblies of high-complexity sequences, using the longer read capabilities of certain next-generation sequencing platforms. For example, we found that we were not able to increase the unique taxa we could detect using reads from the *de novo* assembly method.

All of the identified genera across the treatments will guide the assembly of more complete reference databases for improved taxonomic assignment in future work. The identification of the significant enrichment of *Paenibacillus* (a species known to inhibit and kill *Salmonella*) is an important finding in itself. Researchers attempting to culture *Salmonella* from phyllosphere samples should be aware of this finding. Our results suggest that bioinformatic detection approaches using a combination of metagenomic and shotgun sequence data from classical enrichment methods may be useful both for detection of pathogens and for streamlining of culturing methods.

## Supporting Information

Figure S1 Results from the PCoA based on taxonomic assignments using a subsampled dataset (∼25% of observed data) for both Flashed and Meta-Velvetg. Patterns were consistent across multiple levels and serve to illustrate that our observed pattern is not an artifact of insufficient sampling depth.
(TIFF)
