# Supplementary figures and images for: Co-Enriching Microflora Associated with Culture Based Methods to Detect Salmonella from Tomato Phyllosphere
Source: PLoS One. 2013 Sep 9;8(9):e73079. doi: 10.1371/journal.pone.0073079 (PMC3767688; doi:10.1371/journal.pone.0073079)

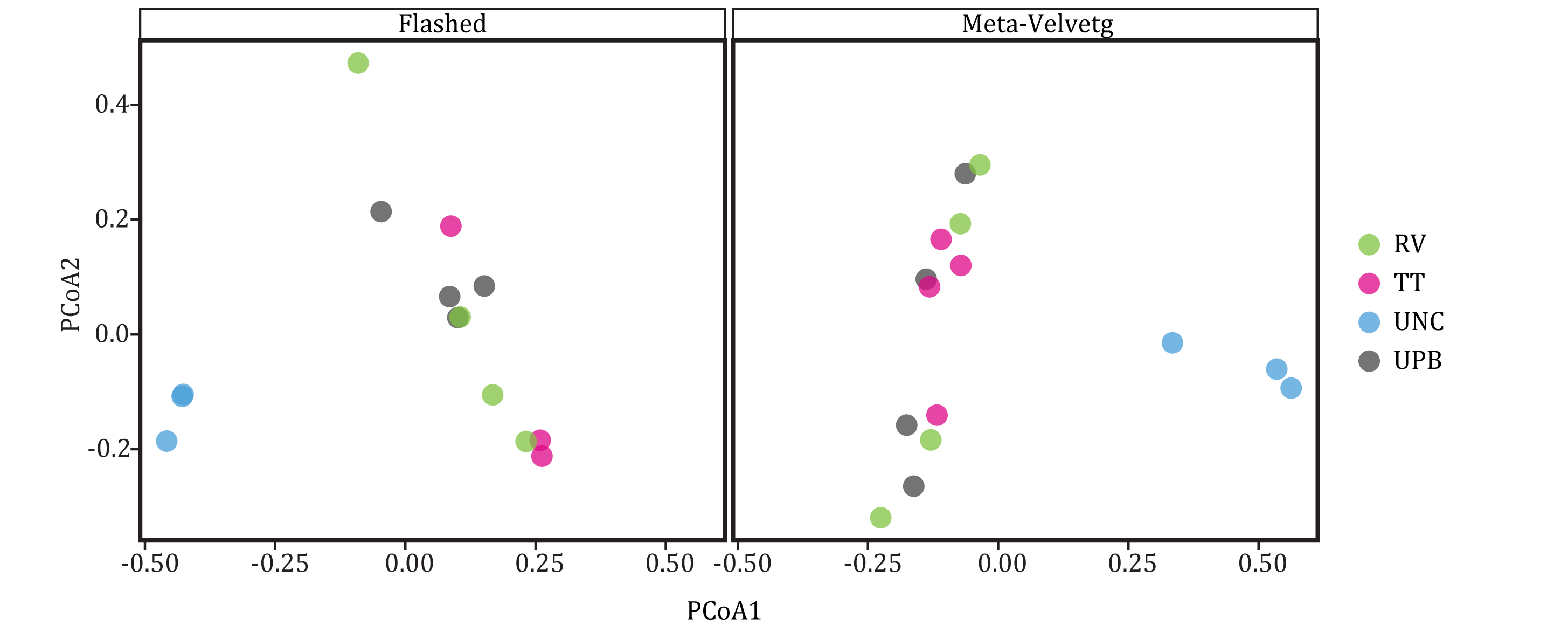

Supplement: Figure S1 — Results from the PCoA based on taxonomic assignments using a subsampled dataset (∼25% of observed data) for both Flashed and Meta-Velvetg. Patterns were consistent across multiple levels and serve to illustrate that our observed pattern is not an artifact of insufficient sampling depth. (TIFF) [file pone.0073079.s001.tiff]
